# Weak Concordance between Fish and Macroinvertebrates in Mediterranean Streams

**DOI:** 10.1371/journal.pone.0051115

**Published:** 2012-12-10

**Authors:** Stefano Larsen, Laura Mancini, Giorgio Pace, Massimiliano Scalici, Lorenzo Tancioni

**Affiliations:** 1 Leibniz Institute of Freshwater Ecology, Berlin, Germany; 2 Department of Environment and Primary Prevention, National Institute of Health, Rome, Italy; 3 Department of Biology, ‘Roma Tre’ University, Rome, Italy; 4 Experimental Ecology and Aquaculture, Department of Biology, ‘Tor Vergata’ University, Rome, Italy; University of Southampton, United Kingdom

## Abstract

Although anthropogenic degradation of riverine systems stimulated a multi-taxon bioassessment of their ecological integrity in EU countries, specific responses of different taxonomic groups to human pressure are poorly investigated in Mediterranean rivers. Here, we assess if richness and composition of macroinvertebrate and fish assemblages show concordant variation along a gradient of anthropogenic pressure in 31 reaches across 13 wadeable streams in central Italy. Fish and invertebrate taxonomic richness was not correlated across sites. However, Mantel test showed that the two groups were significantly, albeit weakly, correlated even after statistically controlling for the effect of environmental variables and site proximity. Variance partitioning with partial Canonical Correspondence Analysis showed that the assemblages of the two groups were influenced by different set of environmental drivers: invertebrates were influenced by water organic content, channel and substratum features, while fish were related to stream temperature (mirroring elevation) and local land-use. Variance partitioning revealed the importance of biotic interactions between the two groups as a possible mechanisms determining concordance. Although significant, the congruence between the groups was weak, indicating that they should not be used as surrogate of each other for environmental assessments in these Mediterranean catchments. Indeed, both richness and patterns in nestedness (i.e. where depauperate locations host only a subset of taxa found in richer locations) appeared influenced by different environmental drivers suggesting that the observed concordance did not result from a co-loss of taxa along similar environmental gradients. As fish and macroinvertebrates appeared sensitive to different environmental factors, we argue that monitoring programmes should consider a multi-assemblage assessment, as also required by the Water Framework Directive.

## Introduction

Human activities have long impaired the natural dynamics of biotic communities in inland waters systems both directly, for example via hydromorphological alteration, pollution, and introduced species, but also indirectly via modification of river catchment from agriculture and urbanization [1], [2], [3], [4]. Running waters are now considered one of the most endangered of all natural ecosystems, [5], [6] with biodiversity loss representing a major threat to their structure and functioning and a challenge for their sustainable management to present and future generations [7], [8], [9]. Moreover, future climate change is also expected to strongly influence river ecosystems [10], [11], with perspectives particularly worrying for catchments draining semi-arid regions such as the Mediterranean [12].

Thus, the increasing degradation of running waters and the accelerating loss of biodiversity have induced an increasing effort into assessing river impairment using different taxonomic groups [13] For example, macroinvertebrates have been used as indicators for detecting: organic pollution [14], changes to hydrologic regime [15], [16], acidification [17] and sediment deposition [18], [19]. Fish have been often associated with changes to catchment land-use patterns, river connectivity [20] and water quality [21].

In EU Countries, use of the multi-assemblage approach has now become an official policy since the Water Framework Directive (WFD) [22] required the classification of river ecological status using four biotic elements as indicators (diatoms, macrophytes, macroinvertebrates and fish). The rational behind this approach is based in the concept of indicator or surrogate communities, which are expected to be representative of other taxa as well [23], [24].

Consequently, a growing number of studies aimed to compare the discriminatory power and the specific sensitivity of different taxonomic groups to environmental degradation [13], [25], [26], [27], [28].

However, compared to Northern and Central Europe, biological indicators for Mediterranean rivers are poorly developed, even though they are recognised as particularly threatened [29], [30]. For example, in Italy only macroinvertebrates have been officially monitored to indicate the biological quality of running waters [31], even though, increasing attention has been paid to implement EU directives for aquatic conservation and management employing riverine fish [4], [32]. Nonetheless, also in view of the specific requirements of the WFD, it is imperative to evaluate similarities and differences in the response of different taxonomic groups to similar stressors gradients. Although, a growing number of studies are investigating patterns of concordance across a wide range of geographical regions [24], studies within the Mediterranean region are still surprisingly few [33], [34], [35]. Concordance is defined as the correlation in assemblage level biodiversity measures between taxonomic groups over a range of localities [24]. Different mechanisms can drive cross-taxon concordance including i) similar response to the same or correlated environmental gradient; ii) co-loss of species along stress gradients; iii) biotic interactions; iv) random sampling of taxa from the regional species pool. However, identifying the main mechanisms involved is complicated by the large spatial extent of concordance studies.

Therefore, in this paper we assessed if the composition and taxonomic richness of two groups frequently used in bioassessment (benthic macroinvertebrates and fish) showed concordant variation along natural and anthropogenic gradients in Mediterranean river basins in central Italy. Moreover, we also appraised whether the observed concordance apparently resulted from biotic interactions and/or similar co-loss of species along environmental gradients. Specifically, we expected macroinvertebrates and fish to be influenced by rather different environmental drivers considering their different body size and life-history attributes. However, considering the strong gradient in anthropogenic influence across locations, and the potential top-down effect of fish on invertebrates, we also expected to observe significant correlation between assemblage level measures.

## Study Area

Environmental and species assemblage data were collected from thirty-one reaches in thirteen wadeable Mediterranean streams in the Province of Rome (Table S2). Data collection was carried out between May 24th–26th and October 4th–3rd 2004–2005, respectively. Each site was visited once and environmental, fish and invertebrates data were collected in each occasion.

**Figure 1:**
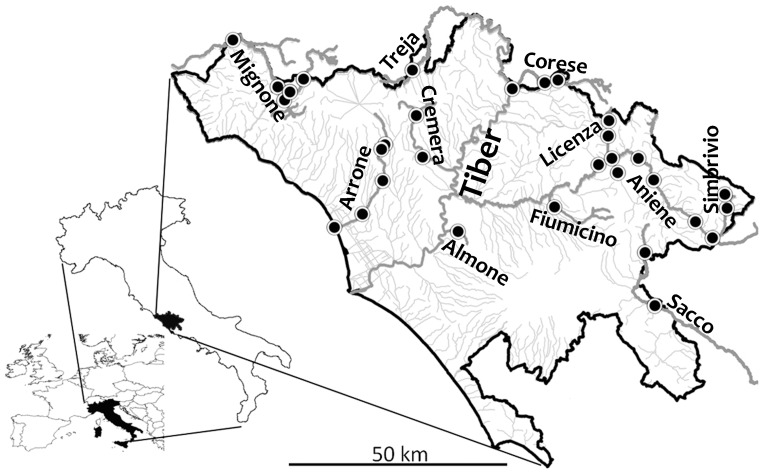
Map of the study area showing the water courses and reaches where fish and macroinvertebrates were collected. Names of the main water courses are reported. See Supporting Information for site coordinates.

Locations covered four river basins (Figure 1; Table S2) including the Arrone (5 study reaches), Liri-Garigliano (2 in the Sacco tributary), Mignone (3 along the river, 2 in tributary Lenta), Tevere (one in Almone, 5 in Aniene, 3 in Corese, 2 in Cremera, 1 in Fiumicino, 3 in Licenza, 1 in San Vittorino, 2 in Simbrivio, 1 in Treja). Reaches were selected to represent the dominant gradient of anthropogenic pressure (i.e. increasing agriculture and settlement) within the region over an altitudinal range of 1–650 m a.s.l., but selection was restricted to reaches where depth (0.3–1 m), flow velocity (0.2–1.0 m/s) and stream width (2–9 m) were as similar as possible.

## Methods

### Environmental data collection

Twenty-seven environmental variables were recorded from each site (Table 1), and categorized in four main descriptors: position in catchment, channel features, substratum features, physico-chemical characteristics and land-use. Physico-chemical and chemical measurements (except for total phosphorous) were collected with a field multi-parameter field probe (Hach – Hydrolab® DataSonde4a; details available at http://www.insight-marine.co.uk/documents/series4a.pdf. Accessed 2012 November 3^rd^). Total phosphorous was measured according to national standards [36]. The geographical information system software (ArcGis; ESRI) was used to quantify land cover within each basin based on the CORINE land-cover database. An Anthropogenic Index (AI) was calculated based on land use information for a 1 km radius around each site as follows:
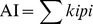
where k_i_ is the specific coefficient for each land-use category and p_i_ is the relative frequency of each category inside the 1-km buffer. The following k values were attributed to the respective CORINE land use categories: 1, natural woods; 2, pastures, meadows, bush areas, scrub and olive grove; 3 agricultural areas and urban green areas; 4, urban and industrial areas. The 1 km buffer was chosen since, in this region, macroinvertebrates appeared influenced by land use at this scale [37], [38]. The index therefore represents a surrogate of anthropogenic development and ranges from 1 (minimum development) to 4 (maximum development).

**Table 1:** List of the measured environmental descriptors.

Environmental variables	units/notes/acronyms
*Position in the catchment*	
latitude	
longitude	
elevation	m a.s.l.
distance from source	Km
*Channel*	*Chan PCA*
depth	m
runs	% of the wetted surface
pools	% of the wetted surface
riffles	% of the wetted surface
flow velocity	cm/s
shade	% of the surface of the sampling site shaded at noon
*Substratum*	*Subs PCA*
boulder	% of the reach surface
cobble	% of the reach surface
gravel	% of the reach surface
silt	% of the reach surface
vegetation cover	% of the reach surface covered by aquatic macrophytes
*physico-chemical*	*P-c PCA*
dissolved oxygen	% saturation
conductivity	µS/cm
ammonium	mg/l
nitrite	mg/l
nitrate	mg/l
turbidity	NTU
chlorofil-a	mg/l
TDS	g/l
total phosphorous	mg/l
*Other physico-chemical*	*Used individually, not in the P-c PCA*
Temperature	
pH	
*Land-use*	
Anthropogenic Index	AI = Σ k_i_ p_i_, where k_i_ is the specific coefficient for each land-use category and p_i_ is the relative frequency of each category inside the 1-km buffer [38].

Channel, substratum and physico-chemical variables were synthesised with Principal Component Analysis. Chan PCA: channel principal components analysis; Subs PCA: substratum principal components analysis; P-c PCA: physico-chemical principal components analysis. AI = Anthropogenic Index.

### Fish and Macroinvertebrate collection

The sampling of animals (fish and invertebrates) was carried out as part of the biological monitoring required for the Regional Fish Biodiversity Data Collection Programme 2005–2009 (ARP Lazio - Regional Parks Agency and Provincia di Roma), under the authorization n. 526425 (Regional Law 87/1990).

Fish were electro-fished by a standard shoulder-bag (4 KW, 0.3–6 Ampere, 150–600 Volt; 0÷100 i/s) according to the national protocols and WFD requirements [32], [39] sampling all available habitats along 40–70 m stream (transect length about 10 times the width of wetted channel). Each specimen was identified, photographed and released at the site. Fish were identified with the aid of a taxonomic guide [40]. All relevant ethical safeguards have been met in relation to animal experimentation. In particular, according to the Italian Guidelines for sampling and analysis of fish fauna of lotic systems [39], all captured fishes were anesthetised with MS 222 solution (Tricaine 92 Methanesulfonate), photographed, and released.

To collect macroinvertebrates, all available habitats within the study reach were initially kick sampled for 3 minutes with a 250 µm mesh net. Samples were first examined in the field, and successive samples were taken until no additional families were found. Protocols followed national standards and no protected species were collected [41].

Samples were then preserved in 70% alcohol and identified in laboratory at genus, subfamily, and family level by using taxonomic guides [42], [43], [44], [45], [46].

### Data analysis

Variables that showed high (>0.7) inter-correlation were omitted. In the present dataset strong correlation occurred between reach elevation and water temperature (r = 0.72) so that analyses were performed using temperature only. Results and conclusion were identical if we used elevation instead.

Proportional data were arc-sin square-root transformed to approximate normal distribution and model assumptions. First of all, principal component analysis (PCA) was used to reduce environmental variables into few independent and interpretable components (PCs). In particular, separate ordinations were performed for: 1) channel variables (Chan PCA; see list); 2) substratum (Subs PCA); 3) physico-chemical variables (P-c PCA). Temperature and pH were not included in the PCA, but were used individually.

Presence/absence data were used for both macroinvertebrates and fish; use of such binary data reduces the errors associated with estimation of species abundance. Fish were identified to species and macroinvertebrates to genus and family level.

Spearman rank correlation was used to compare fish and macroinvertebrate richness across sites.

In order to assess the proportion of variance in taxonomic richness independently explained by each of the explanatory variables, we used hierarchical partitioning of R^2^ values with the HIER-PART package [47] within the R statistical package [48]. This method identifies variables with strong independent correlation with the response variable and those whose eventual correlation with the response variable only result from a joint correlation with other independent variables. Randomization test was used to compare the observed independent contribution of variables to explained variance against a population of independent contributions drawn from 500 randomization of the data matrix. The statistical significance of the variables was determined using the upper 95% confidence limits [49]. This approach is less affected by multi-collinearity between variables and identifies causal/explanatory relationships between treatment and response variables [49].

Canonical Correspondence Analysis (CCA) and partial CCA (pCCA) were used to partition variance in both fish or macroinvertebrates assemblages into unique and shared contribution of environmental and biological variables (see below). In this analysis, the unique contribution of one group in explaining variation in the assemblage of the other group is an estimate of the importance of biological interactions [27]. CCA was chosen for both groups, as gradient length was more than 3.5 in an initial DCA (Detrended Correspondence Analysis), suggesting unimodal species response to environmental gradients [50]. Taxa with less than 3% of occurrence were considered rare and omitted from these analyses to avoid overweighting their influence on ordination results [50], as incidental taxa diminish the response signal of the more abundant taxa to environmental gradients.

Partial CCA enables decomposition of variance [51] and was used to partition the variance into: i) the unique or pure variation explained by environmental variables after removing the (co)variation associated with the other taxonomic group, ii) the pure variation explained by the other taxonomic groups after removing the (co)variation associated with environmental variables, iii) the common or shared variation between environmental and biological variables, and iv) unexplained variation.

First, CCA with no covariables (using both environmental variables and biological parameters from the other group as explanatory variables) was used to calculate the total amount of variance explained. In these analyses the first two DCA axes and taxonomic richness of one group were used as biological explanatory variables when analysing the other group. In a second step, the unique effect of environmental variables or biological variables was estimated by using one as a predictor and the other as a covariable. We used a Monte Carlo permutation test (generating 999 permutations) to test the significance of each environmental variable; permutations were restricted by the covariable matrix in pCCA [25], [51]. All ordinations and permutation tests were performed using CANOCO 4.0 for Windows software [50].

To assess concordance between fish and invertebrates, we compared taxa dissimilarity (Bray-Curtis) matrices using the Mantel test in Past [52]. Partial Mantel test was also used in order to control for environmental variables and site geographical location (using Euclidean distance matrices), as concordance between taxa matrices could derive simply by their shared response to abiotic parameters or by their proximity [53]. Random permutations (5000) were used to obtain the significance level for the correlation coefficients.

As cross-taxon concordance can result from parallel co-loss of species along environmental gradients, we assessed the presence and causes of nestedness in species assemblages.

Nested assemblages occur when species present in species-poor locations represent a subset of the species in richer locations; in other words, in nested systems, rare taxa occur only in the richest sites, while common, more generalist species occur in most locations. While nested species assemblages can result from natural colonization and extinction processes across a fragmented landscape [54], it has been shown that environmental change and human disturbance can also promote nestedness in sensitive organisms [55], [56], [57]. Nestedness is therefore a measure of both richness and composition that, however, have been seldom included in environmental assessment and concordance studies in Mediterranean rivers systems.

Nestedness across locations was assessed using the binary matrix nestedness temperature calculator (BINMATNEST) [58], which is a recent improvement of the nested-temperature method of Atmar and Patterson [59] using a more robust algorithm for matrix packing. This method was chosen as it correlates well with other existing metrics and it is relatively insensitive to matrix size and fill [58]. BINMATNEST reorders the species presence/absence matrix in order to maximize matrix nestedness and then calculates a temperature (ranging over 0–100°C) that represents its deviation from a perfectly nested matrix. Perfectly nested structures with rare taxa only occurring in the richest sites have T = 0°C, while random matrices have T = 100°C.

Statistical significance of the observed temperatures against chance expectation was calculated using Monte Carlo randomisation of 400 simulated matrices. In these simulations, the conservative null-model III was used, where the probability of a cell being occupied equals the average probabilities of occupancy of its row and column; this model is particularly reliable as it is less sensitive to species richness and occurrences [58].

Finally, the order with which the locations are sorted in the maximally packed matrix can be compared with independent variables to assess the likely determinant of nestedness. Spearman rank correlation was used to investigate relationship between nested patterns and environmental parameters for both fish and invertebrate communities. A Bonferroni correction of p-values was applied here, but we provide the exact values of probability since these were very low.

## Results

### Environmental variables

Loadings on the first two Principal Components for the three sets of PCA on channel, substratum and physico-chemical variables are reported in Table 2. PC1 from the channel variables separated reaches characterised by sequences of pools and riffles from wider reaches with run-type flow. PC1 from the substratum features represented a gradient from reaches with fine bed material (sand, silt) to those dominated by cobbles and boulder. Finally, PC1 from the PCA on physico-chemical variables mostly represented a gradient of nutrient enrichment. In all three sets of analyses, the first two PCs explained more than half of observed variation; these two axes were therefore used in subsequent analyses to synthesise reach characteristics.

**Table 2:** Loadings of the first two principal components (PC1 and PC2) of the PCAs on the three sets of environmental variables.

Chan PCA	PC1 (45.2%)	PC2 (31.7%)
runs	−0.97	−0.18
pools	0.90	0.21
riffles	0.78	0.10
width	−0.53	0.73
flow	−0.10	0.68
depth	0.16	0.87
shading	0.30	−0.41

Subs PCA	PC1 (44.1%)	PC2 (24.7%)
boulder	−0.49	0.54
cobbles	−0.83	0.29
gravel	−0.36	−0.80
sand	0.93	−0.16
silt	0.82	0.28
vegetation	0.22	0.61

P-c PCA	PC1 (37.8%)	PC2 (17.9%)
turbidity	0.49	−0.70
conductivity	0.46	−0.05
O_2_% dissolved	−0.52	−0.50
NO_3_^−^	0.69	−0.17
NO_4_^−^	0.68	0.15
NH_4_^+^	0.75	0.35
PO_4_^3−^	0.85	−0.06
TDS	−0.02	0.62
Chl-a	0.66	−0.13

The percentage of explained variation for of each principal component is given in parentheses. Chan PCA: channel principal components analysis; Subs PCA: substratum principal components analysis; P-c PCA: physico-chemical principal components analysis.

The Anthropogenic Index ranged from 1–3.6, meaning that the sampled reaches represented an adequate wide range of landscape development within the region.

### Hierarchical partitioning of taxonomic richness

In total 62 and 31 taxonomical units were identified for macroinvertebrates and fish, respectively (Table S1).

Fish and macroinvertebrate taxonomic richness was not correlated across locations (p = 0.29).

**Figure 2:**
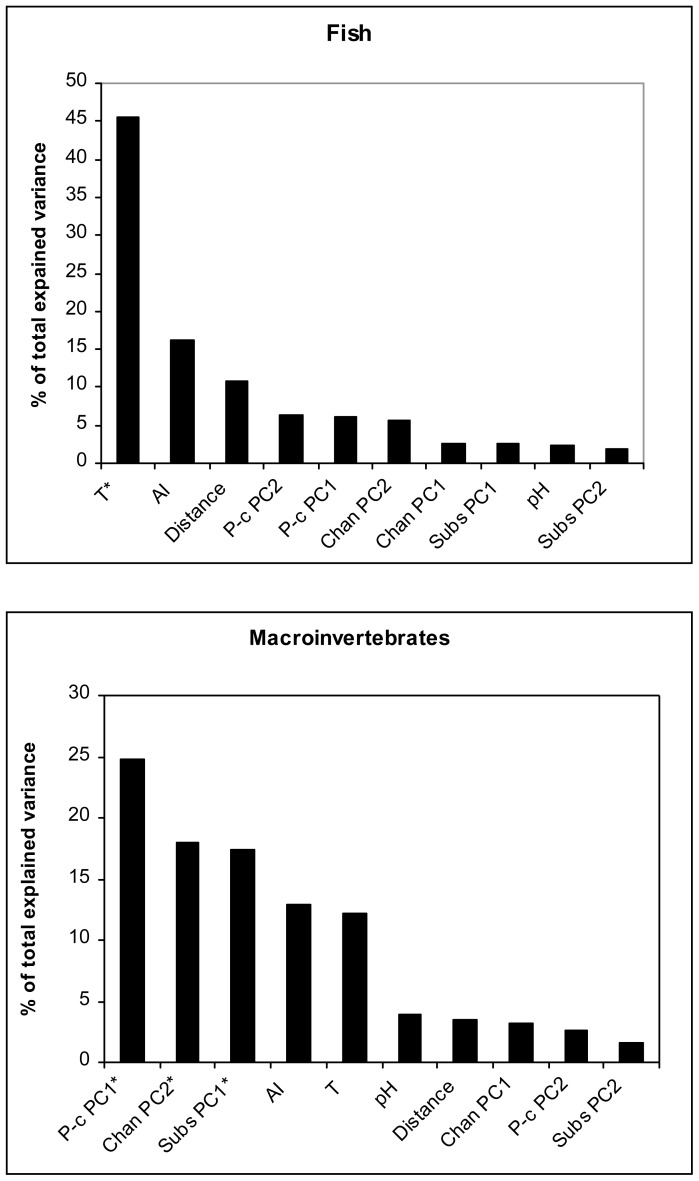
Distribution of independent effects (*I%*) of predictor variables calculated with hierarchical partitioning of fish and macroinvertebrate taxonomic richness. Chan PC: channel principal components; Subs PC: substratum principal components; P-c PCA: physico-chemical principal components; T: temperature; AI: Anthropogenic Index; Distance: distance from source. * denotes statistically significant variables selected by the hierarchical partitioning procedure.

For macroinvertebrates taxonomic richness the variables with the greatest independent explanatory power were Physico-chemical PC1, Channel PC2 and Substratum PC1 (all negatively correlated with richness), each explaining a significant proportion of variance (Table 3; Figure 2). In other words, macroinvertebrate taxonomic richness was lower in reaches with higher nutrient load (nitrates, phosphates, ammonia), and also declined in smaller and shallower reaches and in reaches dominated by fine substrata. The Anthropogenic Index and water temperature, although not significant, also explained a relatively high proportion of variance, and they both correlated negatively with richness.

**Table 3:** Hierarchical partitioning of predictor variables explaining fish and macroinvertebrate richness.

Dependent	Predictor	*I %*	*I*	*J*	Total	*Z-score*	Coefficient
Fish	Temperature	45.49	0.19	0.26	0.45	6.89*	0.65
richness	AI	16.31	0.07	0.04	0.11	0.49	0.38
	Distance from source	10.71	0.04	0.03	0.08	1.19	0.34
	P-c PC2	6.27	0.03	0.05	0.08	0.24	−0.30
	P-c PC1	6.11	0.03	0.05	0.07	0.32	0.27
	Chan PC2	5.61	0.02	0.01	0.03	0.43	0.03
	Chan PC1	2.69	0.01	0.01	0.02	−0.17	0.13
	Subs PC1	2.50	0.01	0.02	0.02	−0.24	0.18
	pH	2.41	0.01	0.01	0.02	−0.35	0.09
	Subs PC2	1.90	0.01	0.02	0.02	−0.53	−0.15
Macroinvertebrate	P-c PC1	24.84	0.12	0.31	0.44	3.78*	−0.69
richness	Chan PC2	17.96	0.09	0.23	0.32	4.14*	−0.54
	Subs PC1	17.37	0.09	0.25	0.34	3.68*	−0.56
	AI	12.91	0.06	0.16	0.22	0.90	0.48
	Temperature	12.13	0.06	0.16	0.22	1.63	−0.48
	pH	3.92	0.02	0.05	0.07	−0.05	0.29
	Distance from source	3.47	0.02	0.04	0.06	0.05	0.18
	Chan PC1	3.25	0.02	0.03	0.04	−0.02	0.15
	P-c PC2	2.57	0.09	0.23	0.32	−0.20	−0.17
	Subs PC2	1.54	0.01	0.00	0.00	−0.41	−0.01

Table shows the independent (*I*), joint (*J*) and total effect of predictors on taxonomic richness. The percent contribution of the predictor to the explained variance of the response variable is shown as *I%*. Z-scores are based on the distribution of randomized *Is* and are calculated as [observed−mean (500 randomizations)]/SD (500 randomizations), and statistical significance (*) is based on the upper 0.95 confidence limit (Z≥1.65). The simple correlation coefficient is also shown to clarify the nature of predictors' relationship with taxonomic richness, but it is not part of the hierarchical partitioning analysis. Acronyms as in Table 1.

For fish, water temperature appeared the most important variable explaining almost half of the total explained variation in richness (Table 3).

### Community composition and variance partitioning

**Figure 3:**
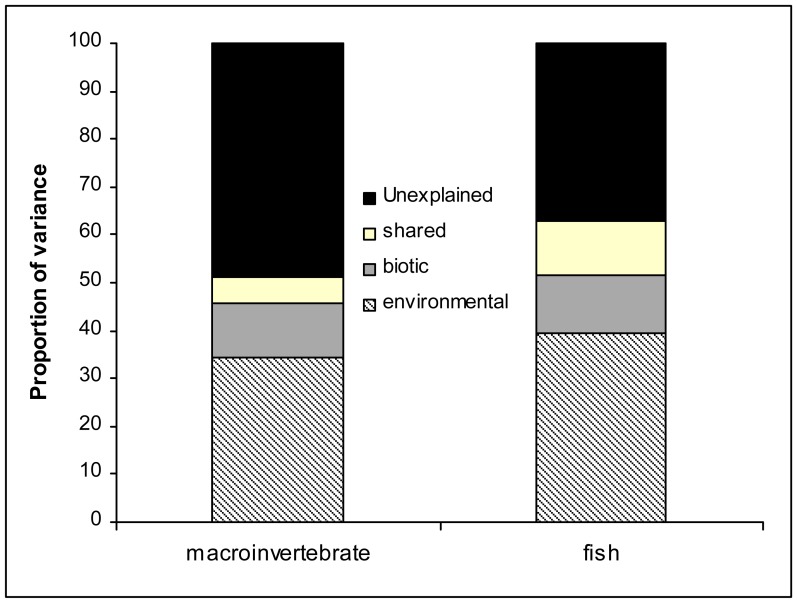
Partitioning of variance in taxonomic composition of macroinvertebrate and fish with partial Canonical Correspondence Analysis showing i) the unexplained variation; ii) the unique effect of environmental variables; iii) the unique effect of biotic variables and iv) the shared effect of environmental and biotic variables. See text for more details.

More than 50% of the variation in macroinvertebrate composition was explained by the initial CCA with both environmental and biotic (i.e. fish DCA axis 1 and 2, and fish richness) variables. The variables identified as significant by Monte Carlo permutations are shown in Table 4. Fish DCA axis 1 appeared as the strongest predictor, explaining the greatest proportion of variation. According to the subsequent partial CCA using biotic variables as co-variables, environmental variables alone (their unique effect) explained 34.5% of variation in macroinvertebrate composition (Table 4 and Figure 3), with the Anthropogenic Index, Physico-chemical PC1 and Channel PC1 explaining a significant proportion of variation. Examining the unique effect of biotic variables, the partial CCA using environmental variables as co-variables showed that more than 11% of macroinvertebrate variation was explained by biotic (fish) variables (corresponding to 22% of the total explained variation). More than 5% of the explained variation could not be attributed to either environmental or biotic variables, being shared between the two.

**Table 4:** Results of forward variable selection in CCA and partial CCA (i.e. the unique effect of environmental variables) performed on macroinvertebrate and fish occurrence matrix.

	Selected variable	Lambda A	P	F
CCA	DCA1 fish	0.29	0.002	2.98
macroinvertebrate (51.3%)	Subs PC1	0.18	0.006	1.95
*(canonical eigenvalue = 1.58)*	AI	0.13	0.05	1.45
partial CCA	AI	0.17	0.004	1.87
macroinvertebrate (34.5%)	Chan PC1	0.12	0.09	1.34
(*canonical eigenvalue = 1.06*)	P-c PC1	0.14	0.038	1.51

	Selected variable	Lambda A	P	F
CCA fish (63.1%)	Temperature	0.47	0.002	4.65
*(canonical eigenvalue = 2.15)*	DCA1 invert	0.29	0.002	3.06
	DCA2 invert	0.23	0.004	2.58
	P-c PC2	0.18	0.016	2.07
	Chan PC2	0.17	0.014	2.06
partial CCA fish (39.5%)	Temperature	0.29	0.002	3.26
*(canonical eigenvalue = 1.34)*	P-c PC2	0.2	0.006	2.36
	Chan PC2	0.19	0.002	2.35

Values in parenthesis show the percentage of explained variance and the sum of canonical eigenvalues. Only significant variables are shown. Lambda A represents the conditional effect (the contribution that each variable bring to the canonical eigenvalues in addition to the variables already selected).

Variance decomposition of fish assemblages showed similar patterns, although the influence of environmental drivers differed. The initial CCA with both environmental and biotic (i.e. macroinvertebrates DCA axis 1 and 2, and taxonomic richness) variables explained 63% of community variation; temperature appeared the most important variable after Monte Carlo permutations, followed by the two macroinvertebrates DCA axes, P-c PC2 and Chan PC2 (Table 4). After controlling for the biotic variables in partial CCA, the unique effect of environmental variables accounted for almost 40% of fish community variation, with temperature explaining the greatest proportion (Table 4). The unique effect of biotic variables accounted for 12% of community variation in partial CCA (corresponding to 19% of the total explained variation), whereas the shared effect of biotic and environmental variables accounted for more than 11% of the variation.

### Community similarity

Bray-Curtis similarity matrices of macroinvertebrate and fish were significantly correlated in Mantel test (r = 0.23; p = 0.001). After controlling for site proximity and environmental variables in partial Mantel tests, the correlation appeared weaker but still significant (r = 0.20; p = 0.004 and r = 0.20; p = 0,038, respectively).

### Nestedness

Both macroinvertebrate and fish community showed significant nestedness in assemblage composition (T = 19.9; p<0.001 and T = 7.5; p<0.001, respectively). Site ranking in the maximally packed macroinvertebrate matrix (i.e. the matrix as ordered by BINMATNEST to maximise nestedness) was significantly positively correlated with Physico-chemical PC1 (r_s_ = 0.62; p<0.001), temperature (r_s_ = 0.52; p = 0.002) and Substratum PC1 (r_s_ = 0.5; p = 0.004). In other words, there was a progressive non-random loss of macroinvertebrate taxa with increasing nutrient enrichment, water temperature and fine bed material.

**Figure 4:**
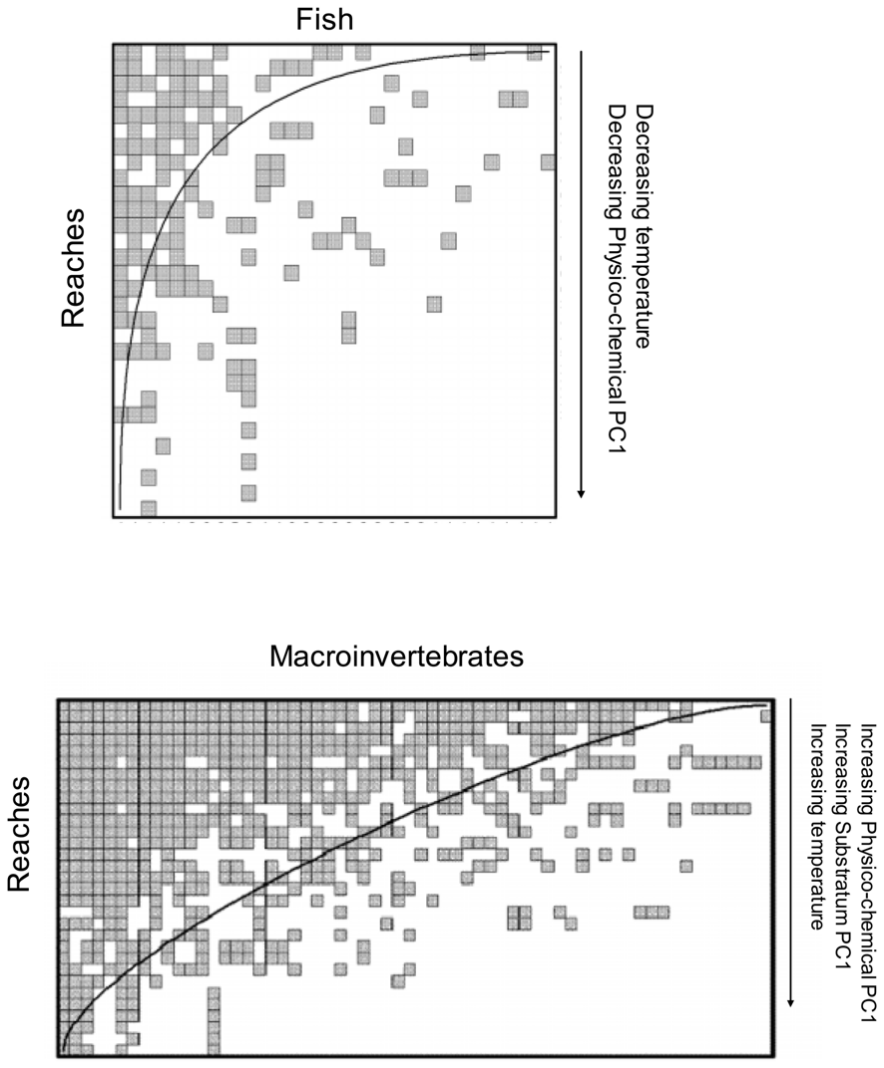
Matrices of fish×reaches and macroinvertebrate×reaches sorted by the software BINMATNEST to maximise nestedness (i.e. minimise unexpected presences and absences). Filled squares represent presence. The curved line shows isoclines of prefect nestedness. Perfect nestedness occurs where rarer species are exclusive to species-rich locations, and where species poor locations host only a subset of species found in richer locations. Arrows represent relationships between the ranking of reaches sorted to maximise nestedness and environmental variables.

For fish instead, the maximally packed matrix appeared correlated negatively with temperature (r_s_ = −0.7; p<0.001) and Physico-chemical PC1 (r_s_ = −0.48; p = 0.005). That is, fish showed an opposite pattern, with a progressive loss of taxa with decreasing temperature and decreasing organic content (Figure 4).

## Discussion

### Taxonomic richness

In agreement with recent studies that observed weak or no relationship among richness of different taxonomic groups [23], [60], [61], macroinvertebrate and fish taxonomic richness was not correlated across the study reaches. This seems to be a common finding in biodiversity studies and Wolters *et al.* [61], in a meta-analysis covering 43 taxa, concluded that no taxon appeared to be a good predictor of the richness of other taxa. The lack of strong correlations is generally attributed to taxon specific responses to environmental gradients [62], and this appears to be the case also in these Mediterranean catchments. Richness of benthic invertebrates was mostly influenced by water quality, declining with increasing phosphates and nitrates, as reflected by the physico-chemical PC1. In addition, smaller reaches and those dominated by fine bed material also supported lower macroinvertebrate richness. These results are not surprising and are in agreement with numerous studies reporting similar patterns in this region and elsewhere [19], [63], [64], [65].

Fish richness instead appeared to largely follow the temperature (and altitude) gradient with warmer locations at lower altitudes supporting more species. This could reflect the natural longitudinal distribution of fish species in the study catchments, where higher temperatures downstream allow for the co-occurrence of more species, even within a relatively small altitudinal range. Indeed, the longitudinal pattern of increasing fish species richness is commonly observed in streams and rivers in both temperate and Mediterranean catchments [66], [67], [68], [69].

Therefore, macroinvertebrate and fish taxonomic richness appeared to be governed by different environmental gradients. However, taxonomic richness at the site is just one currency of biodiversity and, in testing between-taxa congruence, patterns and similarity in assemblages also needs to be addressed.

### Community assemblages and similarity

Results from constrained ordinations and variance partitioning confirmed the finding that the two assemblages were influenced by different environmental drivers. In the full CCA models (including environmental and biological variables) of both fish and invertebrates the total explained variation was relatively high for field investigations, explaining more that 50% [27] and indicating that the variables considered were indeed those influencing community structures. Variance partitioning with partial CCA indicated that benthic invertebrate assemblages were predominantly influenced by local land-use (as expressed by the Anthropogenic Index), channel morphology and water quality, each explaining a significant proportion of variance. Previous surveys support the role of local land-use and morphological features in structuring benthic assemblages in this Region [37], [38], [70].

Fish assemblages, instead, appeared to follow the temperature (and altitudinal) gradient, which alone accounted for the greatest proportion of variance explained. This result is expectable and likely reflects the longitudinal change in fish assemblage commonly observed in lotic systems [67], [69], and the significant influence of channel depth and width (as expressed by Channel PC2) on fish assemblages further supports this view. In fact, this observation agrees with the longitudinal fish zonation with dominance of few native species in the upstream water courses and higher number of taxa in downstream reaches [4], [71], [72]. More interestingly, however, the analyses revealed the importance of biological interactions in shaping the communities. For both benthic invertebrates and fish, taxonomic composition of the “other” group (expressed as DCA axes) was selected as one of the most important explanatory variables, in each case accounting for ∼20% of the total explained variation. Clearly, biotic interactions between fish and macroinvertebrates are complex and could only be indirectly inferred in this study, so that specific investigations and experiments are needed to clarify their nature. Nonetheless, these interactions can promote concordance among freshwater groups that otherwise show different environmental sensitivity. For example Johnson and Hering [25] showed that in semi-natural streams, the composition of other co-occurring taxonomic groups was a better predictor of assemblage composition than environmental characteristics alone. On the same theme, Jackson and Harvey [73] observed similar structure between fish and invertebrates across lakes, but different relationship between each taxonomic group and lakes' environmental conditions; an apparent paradox that could be explained by strong biotic interactions between the groups.

Further evidence of potential biotic interactions in our study derives from partial Mantel tests, in which fish and invertebrate assemblages showed significant, albeit weak, concordance even after removing the effect of environment and geographical distance. This means that the association between the two groups does not result from geographical proximity or similar response to environmental gradients. In this case, our results parallel those of Grenouillet *et al.* [53] who observed significant congruence among fish and invertebrate along the River Viaur after controlling for longitudinal and environmental distance among locations; a finding that was attributed to direct trophic interactions, although these could not be demonstrated in the field.

### Nested species assemblages

Although nestedness is a pattern often observed in species distribution [23], [56], [74], [75], few studies have simultaneously assessed it for different taxonomic groups [76], [77]. Moreover, as concordance between assemblages can also result from similar species loss along environmental gradients, it is surprising that nestedness is seldom investigated in concordance studies [78], [79].

We found that both macroinvertebrate and fish assemblages were significantly nested across the study reaches. Clearly - as in any ecological study - nestedness was far from perfect, especially for macroinvertebrates, whereas fish assemblages showed relatively low matrix temperature (i.e. higher nestedness).

Different mechanisms appeared to promote nestedness in the two groups. For macroinvertebrates, stream reaches with deteriorating water quality, increasing temperature and fine sediments supported only a sub-set of taxa present in richer locations. This result suggests that environmental gradients may act as filters for community assembly, progressively selecting for those taxa tolerant or adapted to the predominant environmental conditions. The degree of nestedness and its potential drivers are in line with previous studies on stream invertebrates. Both Heino *et al.* [80] and Larsen and Ormerod [57] observed that environmental characteristics such as water quality and substratum structure were correlated with patterns of nestedness in aquatic invertebrate communities. In particular, our results are in agreement with those of Larsen and Ormerod [57] in showing that reaches dominated by fine bed material were associated with the formation of nested assemblages, likely as a result of substratum homogenisation.

Nested pattern in fish communities, instead, appeared predominantly influenced by the temperature gradient, with colder water reaches mostly supporting a sub-set of fish species present in warmer and richer locations. This reflects the progressive non-random loss of species with decreasing water temperature (and increasing altitude), which characterises the longitudinal fish distribution in the study catchments. In fact, this is an expectable finding, and Cook *et al.* [81] also observed a positive correlation between elevation and the degree of nestedness of fish communities in Virginia. They concluded that habitat factors associated with elevation, such as temperature and productivity ultimately determined fish species occurrence.

More importantly however, these results show that the apparent mechanisms behind the nested distribution of taxa differed between macroinvertebrates and fish. This observation rules out the possibility that the (weak) concordance observed between the two groups could derive from parallel drop-out of species along the same environmental gradient. Moreover, our results corroborate those from other studies investigating nestedness across taxonomic groups. Although nestedness appeared to be a commonly observed pattern, the apparent mechanisms behind its formation differ among different groups, including for instance birds, butterfly, lizards and small mammals [76], [77], [82]. The differences in mechanisms influencing nestedness is often related to variation in life-history traits among taxa, as well as habitat and area requirement. We observed no effect of area (stream size) on nestedness of either fish or macroinvertebrates, but results suggest that other habitat related factors may dictate extinction and colonization dynamics in these Mediterranean catchments.

These results have also important environmental management implications. Since the mechanisms influencing nestedness differ between the two study groups, caution is needed in using fish and invertebrates as surrogate of each other in sustainable management planning.

## Conclusion

The present study investigated pattern of concordance between two groups widely used in freshwater bio-assessment, such as macroinvertebrates and fish. Current knowledge on taxonomic congruence among Mediterranean freshwater groups is still relatively poor [34], [83], and to our knowledge no such study has been documented within the Italian region.

As previously explained, between-taxon congruence mainly results from few non-mutually exclusive mechanisms including i) similar response to the same or correlated environmental gradients; ii) co-loss of species along stress gradients; iii) biotic interactions; and iv) random sampling of taxa from the regional species pool. Although identifying responsible mechanisms is challenging, we were able to partly clarify the nature of fish - invertebrate concordance in these Mediterranean streams. First, although taxonomic richness of the two groups was not correlated, assemblages showed significant, albeit weak (namely<0.7) concordance, dissuading the use of one taxa as surrogate of the other.

Second, rather different environmental gradients appeared to influence fish and macroinvertebrate occurrence; nutrient enrichment and stream channel features chiefly determined macroinvertebrate richness and composition, while fish assemblages and richness appeared to mainly follow the temperature and elevation gradient.

Third, partial ordination and Mantel tests revealed the potential importance of biotic interactions as driver of community concordance. Although the specific nature of such interactions (e.g. predation) still remain to be demonstrated in our study streams, top-down effects of fish on invertebrate communities have been widely observed [84], [85], [86].

Forth, mechanisms regulating local extinction – colonisation dynamics also differed for the two groups, as showed by the nestedness pattern analysis. These considerations suggest that the two groups provided rather complementary ecological information and that eventual conservation measures must be implemented taxon-specifically.

Because streams and rivers are usually affected by multiple and inter- acting disturbances [6], [11], and biological responses could be scale-dependent, studies of this kind can provide key information on the relative sensitivity of different indicator groups.

Nonetheless results from this study must be interpreted with caution and some limitations must be acknowledged. For example, while estimates of abundance or biomass for the two groups were not available, there is evidence that often biomass values are more strongly correlated across taxa than richness or assemblage composition [60]. Moreover, both fish and invertebrates communities can show large seasonal variability, especially in Mediterranean areas [87], [88], so that conclusions drawn from one sampling occasion ought to be considered preliminary. In addition, while results could also have been influenced by the different taxonomic resolution utilised for fish and macroinvertebrates, the latter are notably difficult to identify at the species level. However, several studies have shown that general community patterns holds across different taxonomic resolution levels [89], [90], and that strong correlation is often observed between species number and taxon number at coarser resolutions not only in aquatic systems [90], [91], [92].

Overall however, this work is in line with recent studies in showing weak concordance and the limited value of surrogacy in freshwater systems [60], [93]; on the other hand, results also support the requirements of the European Water Framework Directive in the need of simultaneously analysing different biological elements (taxonomic groups) to assess ecological status of aquatic ecosystems.

## Supporting Information

Table S1 Collected taxa listed alphabetically.
(DOC)

Table S2 Names and coordinates (decimal degrees) of each study site in the four catchments.
(DOC)
