# Supplementary material for: Weak Concordance between Fish and Macroinvertebrates in Mediterranean Streams
Source: PLoS One. 2012 Dec 10;7(12):e51115. doi: 10.1371/journal.pone.0051115 (PMC3519485; doi:10.1371/journal.pone.0051115)
Supplement: Table S1 — Collected taxa listed alphabetically. (DOC) [file pone.0051115.s001.doc]

Table S1

| Macroinvertebrate | | Fish |
| --- | --- | --- |
| Ancylidae | Odontoceridae | *Alburnus arborella* |
| Asellidae | *Onychogomphus sp.* | *Anguilla anguilla* |
| Astacidae | Palaemonidae | *Barbus plebejus* |
| Athericidae | *Perla sp.* | *Barbus tyberinus* |
| *Baetis sp.* | Philopotamidae | *Carassius auratus* |
| Beraeidae | Physidae | *Carassius carassius* |
| Bythiniidae | *Pisidium sp.* | *Protochondrostoma genei* |
| *Caenis sp.* | Polycentropodidae | *Clarias gariepinus* |
| *Calopteryx* | *Protonemura sp.* | *Cobitis bilineata* |
| Ceratopogonidae | *Rhithrogena sp.* | *Cyprinus carpio* |
| Chironomidae | Rhyacophilidae | *Dicentrarcus labrax* |
| *Choroterpes sp.* | Sericostomatidae | *Gambusia holbrooki* |
| *Dina sp.* | *Serratella sp.* | *Gasterosteus aculeatus* |
| *Dinocras sp.* | Sialidae | *Gobio gobio* |
| Dryopidae | Simulidae | *Padogobius nigricans* |
| *Dugesia* | Sphaeriidae | *Ictalurus punctatus* |
| Dytiscidae | Stratiomidae | *Lampetra planeri* |
| *Ecdyonurus sp.* | Tabanidae | *Lepomis gibbosus* |
| Elmidae | Tipulidae | *Leuciscus cepahlus* |
| *Epeorus sp.* | Tubificidae | *Leuciscus lucumonis* |
| Gammaridae |  | *Leuciscus souffia muticellus* |
| *Gonphus sp.* |  | *Liza ramada* |
| Gordiidae |  | *Mugil cephalus* |
| *Harophlebia sp.* |  | *Padogobius bonelli* |
| *Helobdella sp.* |  | *Pseudorasbora parva* |
| *Heptagenia sp.* |  | *Rutilus erythrophthalmus* |
| Hydraenidae |  | *Rutilus rubilio* |
| Hydrobioidea |  | *Rutilus rutilus* |
| Hydrophilidae |  | *Salaria fluviatilis* |
| Hydropsychidae |  | *Salmo trutta* |
| *Isoperla sp.* |  | *Scardinius erythrophthalmus* |
| Lepidostomatidae |  |  |
| Leptoceridae |  |  |
| *Leuctra sp.* |  |  |
| Limnephilidae |  |  |
| Limoniidae |  |  |
| Lumbricidae |  |  |
| *Lymnaea sp.* |  |  |
| Lymnaeidae |  |  |
| Nepidae |  |  |
| Neritidae |  |  |
| Notonectidae |  |  |

Collected taxa listed alphabetically.
