# Supplementary material for: Weak Concordance between Fish and Macroinvertebrates in Mediterranean Streams
Source: PLoS One. 2012 Dec 10;7(12):e51115. doi: 10.1371/journal.pone.0051115 (PMC3519485; doi:10.1371/journal.pone.0051115)
Supplement: Table S2 — Names and coordinates (decimal degrees) of each study site in the four catchments. (DOC) [file pone.0051115.s002.doc]

| Site | Latitude | Longitude | Catchment |
| --- | --- | --- | --- |
| Arrone1 | 42.035825 | 12.311418 | Arrone |
| Arrone2 | 42.026339 | 12.301503 | Arrone |
| Arrone3 | 41.964051 | 12.307997 | Arrone |
| Arrone4 | 41.897289 | 12.256636 | Arrone |
| Arrone5 | 41.869236 | 12.182921 | Arrone |
| Sacco1 | 41.837084 | 13.012303 | Liri-Gagliano |
| Sacco3 | 41.712801 | 13.091161 | Liri-Gagliano |
| Lenta1 | 42.231008 | 11.894751 | Mignone |
| Lenta2 | 42.142017 | 12.018574 | Mignone |
| Mignone1 | 42.159047 | 12.087688 | Mignone |
| Mignone2 | 42.133147 | 12.052516 | Mignone |
| Mignone3 | 42.231008 | 11.894751 | Mignone |
| Almone1 | 41.868167 | 12.512244 | Tiber |
| Aniene1 | 41.871678 | 13.184153 | Tiber |
| Aniene2 | 41.899886 | 13.144778 | Tiber |
| Aniene3 | 41.979548 | 13.031039 | Tiber |
| Aniene4 | 42.022159 | 12.989711 | Tiber |
| Aniene5 | 42.007853 | 12.882486 | Tiber |
| Cremera1 | 42.094721 | 12.391917 | Tiber |
| Cremera2 | 42.012867 | 12.412570 | Tiber |
| Corese1 | 42.171868 | 12.768387 | Tiber |
| Corese2 | 42.166745 | 12.733808 | Tiber |
| Corese3 | 42.152671 | 12.646589 | Tiber |
| Fiumicino2 | 41.992531 | 12.934938 | Tiber |
| SanVittorino1 | 41.922142 | 12.767649 | Tiber |
| Licenza1 | 42.094439 | 12.907158 | Tiber |
| Licenza2 | 42.066389 | 12.906654 | Tiber |
| Licenza3 | 42.021067 | 12.917739 | Tiber |
| Simbrivio1 | 41.928150 | 13.228306 | Tiber |
| Simbrivio2 | 41.955193 | 13.221997 | Tiber |
| Treja1 | 42.183928 | 12.379172 | Tiber |

Table S2. Names and coordinates (decimal degrees) of each study site in the four catchments.
